# Chloride of Lime in Leucorrhæa

**Published:** 1847-05

**Authors:** 


*Chloride of Lime in Leucorrhœa.*—By MOORE HOIT, M. D.

Sir :—If in your opinion the following trifles deserve a place in a "record of practical medicine," they are at your service :

For several years I have been in the practice of using for this disease, a weak solution of Chloride of Lime as an injection, with the most gratifying success. In that atonic condition of the parts, the result of this disease, or of abuse, or both, in which the womb lies low in the vagina, as though it lacked the support of this organ, I think this remedy will be found to afford more relief than any that, to my knowledge, has ever been proposed. For the Pruritus sometimes attending this disease, and which is so frequent and so distressing after the cessation of the catamenia, it has never as yet disappointed me. The solution I use is made by infusing for an hour, half an ounce of the chloride in a quart of cold water.—*Ibid.*

